# The risk of HIV transmission at each step of the HIV care continuum among people who inject drugs: a modeling study

**DOI:** 10.1186/s12889-017-4528-9

**Published:** 2017-07-25

**Authors:** Daniel J. Escudero, Mark N. Lurie, Kenneth H. Mayer, Maximilian King, Sandro Galea, Samuel R. Friedman, Brandon D. L. Marshall

**Affiliations:** 1 Department of Epidemiology, Harvard T.H. Chan School of Public Health, 677 Huntington Ave, Boston, MA USA; 2 Department of Epidemiology, Brown University School of Public Health, 121 South Main Street (Box G-S-121-2), Providence, RI USA; 3 Fenway Health, 1340 Boylston St, Boston, MA USA; 4 Beth Israel Deaconess Medical Center, 330 Brookline Ave, Boston, MA USA; 5 Boston University School of Public Health, Boston, Albany St, MA 715 USA; 6 National Development and Research Institutes, 71 West 23rd St, New York, NY USA

**Keywords:** People who inject drugs, HIV care continuum, HIV care cascade, HAART, ART

## Abstract

**Background:**

People who inject drugs (PWID) are at continued risk for HIV in the U.S., and experience disparities across the HIV care continuum compared to other high-risk groups. Estimates of the risk of HIV transmission at each stage of the care continuum may assist in identifying public health priorities for averting incident infections among PWID, in addition to transmissions to sexual partners of PWID.

**Methods:**

We created an agent-based model simulating HIV transmission and the HIV care continuum for PWID in New York City (NYC) in 2012. To account for sexual transmission arising from PWID to non-PWID, the simulation included the entire adult NYC population. Using surveillance data and estimates from the National HIV Behavioral Surveillance system, we simulated a dynamic sexual and injecting network. We estimated the proportion of HIV transmission events attributable to PWID in the following categories, those: without an HIV diagnosis (‘Undiagnosed’); diagnosed but not on antiretroviral therapy (ART) (‘Diagnosed − not on ART’); those who initiated ART but were not virally suppressed (‘Unsuppressed’); and, those who achieved viral suppression (‘Suppressed’).

**Results:**

We estimated HIV incidence among PWID to be 113 per 100,000 person-years in 2012, with an overall incidence rate for the entire adult NYC population of 33 per 100,000 person-years. Despite accounting for only 33% of the HIV-infected PWID population, the Undiagnosed were associated with 52.6% (95% simulation interval [95% SI]: 47.1–57.0%) of total transmission events. The Diagnosed − not on ART population contributed the second-largest proportion of HIV transmissions, with 36.6% (95% SI: 32.2–41.5%). The Unsuppressed population contributed 8.7% (95% SI: 5.6–11.8%), and Suppressed 2.1% (95% SI: 1.1–3.9%), relatively little of overall transmission.

**Conclusions:**

Among PWID in NYC, more than half (53%) of transmissions were from those who were unaware of their infection status and more than 36% were due to PWID who knew their status, but were not on treatment. Our results indicate the importance of early diagnosis and interventions to engage diagnosed PWID on treatment to further suppress population-level HIV transmission. Future HIV prevention research should focus on the elimination of identified and potential barriers to the testing, diagnosis, and retention of PWID on HIV treatment.

**Electronic supplementary material:**

The online version of this article (doi:10.1186/s12889-017-4528-9) contains supplementary material, which is available to authorized users.

## Background

The Centers for Disease Control and Prevention (CDC) estimates that about 1 in 11 new HIV diagnoses in the United States (U.S.) occur among those with a history of injection drug use [1]. In recent years, settings such as New York City (NYC) and Vancouver have achieved dramatic reductions in HIV incidence among people who inject drugs (PWID), through efforts including needle and syringe programs and HIV treatment scale-up [2], indicating progress for HIV prevention in high-resource settings. Despite this progress, recent outbreaks have illustrated the need for expanded efforts to prevent HIV transmission among PWID [3, 4], particularly as the use of some injection drugs, such as heroin, has increased in the U.S. [5, 6].

A crucial element of HIV prevention for PWID is the use of antiretroviral therapy (ART) in reducing the infectiousness of HIV-infected individuals [7], in what is known as treatment as prevention (TasP). Reduced individual- and population-level infectiousness have translated to profound reductions in HIV incidence among PWID in many settings [8, 9]. For TasP-based strategies to be fully effective, however, barriers to achieving viral suppression must be eliminated [10, 11]. In recent years, a heuristic has evolved, described as the “HIV care cascade” or “continuum”, which delineates the steps needed to transform an HIV-infected population from being treatment-naïve and infectious to virologically suppressed on highly active antiretroviral therapy, wherein they are functionally unlikely to transmit HIV to new partners [12].

Prior research has estimated that PWID in the earlier stages of HIV care (e.g., undiagnosed, as well as diagnosed but not on ART) might contribute significantly higher rates of transmission (as much as 10-fold), even when compared to those with ineffective or inconsistent treatment [13]. These results suggest that, although achieving viral suppression is the ultimate objective from both an HIV prevention and individual health perspective, improved diagnosis and treatment enrollment may have benefits for population-level reductions in HIV transmission, even without significant improvement in virologic suppression.

Although the estimated HIV incidence among PWID in NYC has declined substantially in recent years [14], due in part to the success of harm reduction (e.g. availability of sterile syringes) and TasP-based strategies [2], gaps in the care continuum may play an important role in ongoing new transmissions observed in this population. Estimating the stage(s) in the care continuum at which these HIV transmission events arise may provide critical information for optimizing the care continuum in NYC and other settings with persistent HIV incidence among PWID.

Using an agent-based model of the NYC population in 2012 (i.e., an individual-based microsimulation of the city’s residents), we provided the first estimate of the contribution of transmission arising from PWID at each step of the HIV care continuum. To account for all transmission events arising from PWID (including those to their sexual partners, who may or may not inject drugs), our model included a representation of the entire adult population, including significant sexual risk groups, such as men who have sex with men (MSM).

## Methods

### Modeling the HIV care continuum

The HIV care continuum, described as comprising five distinct steps along the path from untreated HIV disease towards viral suppression [15], includes those: (1) who are HIV-infected but undiagnosed, (2) who have been diagnosed but have not been referred to, or retained in, medical care, (3) who are retained in care but not prescribed ART, (4) who are prescribed ART but not virally suppressed, and those (5) who are virally suppressed (i.e., having a viral load <200/mL). Given current efforts emphasizing the immediate treatment of all diagnosed persons [16, 17], we collapsed the second and third steps described above into a single step comprised of those with diagnosed yet untreated HIV infection. Thus, we sought to estimate the contribution of transmission from the following four steps in the HIV care continuum, those who are: undiagnosed, diagnosed but not on ART, on treatment but not suppressed, and suppressed.

### Data sources

Where possible data sources were used to estimate the state of the HIV epidemic (e.g., HIV prevalence, incidence) within NYC at the end of 2011, at which point the model simulated the adult NYC population over a one-year period (2012). Inclusion of all city residents (not only PWID) within the transmission network was critical in replicating the true risk environment for PWID (i.e., to avoid erroneously generating a risk network populated by only high-risk PWID), and to account for sexual transmission to non-PWID.

Prevalence and frequency of HIV risk behaviors were estimated using an array of empirical studies and reports among PWID, MSM, and the general population; most notable among these were reports published by the National HIV Behavioral Surveillance (NHBS) system conducted by the CDC [18–20]. Briefly, the NHBS consists of rotating, annual cycles of surveillance among PWID, MSM and high-risk heterosexuals among a total of 22 U.S. cities since 2003 [21].

The number of PWID (defined as those currently engaging in injection drug use [IDU]) in NYC was calculated using HIV prevalence estimates stratified by sex, as well as the total estimated HIV infections among male and female PWID in NYC [22, 23]. The prevalence of MSM in the model was determined using estimates published in 2011 using the NYC Department of Health and Mental Hygiene’s (DOHMH) Community Health Survey [24]. The overall NYC population was assumed to be 50% female, both males and females were categorized by their sex at birth, and all males not categorized as MSM were assumed to be heterosexual.

To inform our estimates for the proportion of PWID in each of the four care continuum steps, we used data presented by the HIV Health and Human Services Planning Council of the NYC DOHMH [25], as well as the NYC-specific data collected by the IDU-3 version of the NHBS [20, 22].

HIV prevalence among PWID, MSM, heterosexual men, and heterosexual women were estimated from data reported by the NYC DOHMH [22, 23], and supplemented by the NHBS to account for undiagnosed (therefore unreported) HIV infection within each subpopulation [20]. Table 1 presents model inputs for selected demographic and HIV treatment related values within the baseline simulated population.

**Table 1:** Baseline Demographic, HIV Prevalence, and HIV Treatment Parameters for Simulated New York City Population, 2012

Variable	Population				
	MSM	HM	Female	PWID^a^	Source
Demographics					
Total population size (%)	5.00	45.00	50.00	2.67	Calculated, [24, 49, 50]
HIV prevalence (%)	22.00	0.30	0.90	17.0 (M), 22.3 (F)	Calculated, [22, 23]
HIV Treatment Parameters					
Proportion diagnosed at baseline	0.77	0.86	0.86	0.61 (M), 0.78 (F)	[19, 22, 25, 51]
Proportion of those diagnosed on ART at baseline	0.57	0.63	0.63	0.55	[25]
Proportion achieving viral suppression^b^	0.84	0.76	0.76	0.69	[25]

*Abbreviations*: *ART* antiretroviral therapy, *HIV* human immunodeficiency virus, *PWID* people who inject drugs

Note: These parameters values apply to the adult population simulated for this analysis, defined as aged >19 years

Note: HIV prevalence within each subpopulation as well as the overall PWID population size were calculated using surveillance estimates obtained from the New York City Department of Health and Mental Hygiene and estimates of population size, as described in the *Estimating HIV Prevalence and Incidence* section of the Additional file 1

^a^Where estimates differ among male and female PWID, they are indicated by an “M” and “F”, respectively

^b^Viral suppression is defined as having a viral load measurement of <200 copies/mL

The frequency of sex acts and condom usage, as well as the number of sexual and injection partners among each of the subpopulations was estimated using NHBS data, from NYC where available, otherwise from the entire study sample, as well as results from previously published sexual behavior studies in NYC and nationwide [19, 22, 26–29].

For PWID, the frequency of injection acts, number of partners, and probability of engaging in receptive needle-sharing was estimated from studies of social/risk networks among PWID in NYC and the U.S., and the NHBS [30–32]. Other data sources used to estimate model parameters can be found in Additional file 1: Table S1.

### Model description

We constructed an agent-based model that generated a representative sample population of 250,000 agents characterizing the demographics and risk-behavior of the NYC population over 19 years of age in 2012. Complete details on the model structure are available in the Additional file 1. In brief, agents represent individuals with demographic, behavioral, and clinical features determined by the distribution of these characteristics in the NYC source population. At each discrete time step, agents form dyads or clusters through which HIV transmission can occur. These links may represent a sexual relationship, an injecting relationship (for PWID), or both, for a given one-month period. After each month, agents may experience partner turnover, in which they may gain or lose partners (see *Network Structure* in Additional file 1).

HIV transmission is possible between any HIV-discordant pair of agents that engage in risk activity. The probability of HIV transmission is determined by: (1) the type of risk behavior (heterosexual intercourse, same-sex activity, or injection); (2) the infected agent’s HIV disease stage (e.g., acute, chronic, AIDS); (3) the infected agent’s ART treatment status; and (4) condom use (if sexual). Furthermore, ART treatment status is stratified by level of adherence, which determines the infectiousness of the HIV-infected agent and thus the likelihood of transmission. These transmission probability estimates can be found in Additional file 1: Table S2, alongside estimates for the probability of progression to AIDS for those of a given ART treatment and adherence level.

Whenever a transmission event occurred, the model catalogued the event by the type of risk act (e.g., sexual or injection), and the HIV care continuum step in which the infected agent belonged. This allowed us to estimate the number and proportion of transmission events that arose from those in each care continuum step. We also calculated the ratio of transmission events in 2012 to the average population size in each care continuum step.

### Movement within the HIV care continuum

We assumed all agents in the model could move along the care continuum in either direction, with the exception of a positive diagnosis, which is always retained. The baseline probabilities of seeking an HIV test in 2012 were first estimated for each subpopulation using self-reported data from the NHBS and elsewhere [19, 22, 24, 33]; these values were then subject to calibration based on new diagnoses reported to the NYC DOHMH (see Additional file 1). Upon receiving an HIV diagnosis, PWID and other agents may initiate ART. The rate of ART initiation was estimated from empirical data [34]. Agents on ART may discontinue therapy at any time, based on previously published literature (references in Additional file 1). The proportion of individuals attaining viral suppression (viral load <200/mL) was estimated for each subpopulation based on levels of suppression reported by the NYC DOHMH in 2011 [25]. Those with partial or no adherence were assumed to be equally distributed among four partial adherence categories (see *HIV Disease Progression and Treatment* in Additional file 1).

### Model calibration and simulations

In some instances, initial model parameters were adjusted during calibration procedures to match empirical estimates (i.e., HIV incidence, HIV diagnoses, levels of viral suppression) within both the general and PWID populations (see *Model Calibration* section and Additional file 1: Table S3).

After calibration, we obtained results representing the mean value of 10,000 Monte Carlo runs. For results on transmission within each care continuum step we calculated 95% simulation intervals representing output values between the .025 and .975 percentiles. Each model run represents one sampling of the study population in 2012, and correspondingly, each run lasted for 12 months.

### Sensitivity analyses

Sensitivity analyses were performed on factors hypothesized to strongly influence the main results. Specifically, we conducted four series of one-way sensitivity analyses, where the following were adjusted: the reduction in PWID risk behavior following a positive HIV test (from a reduction of 25% to reductions of 0% and 50%); the change in agent risk behavior following ART initiation (from no change to a 50% increase and a 50% reduction); the level of viral suppression (from approximately 38% of those with an HIV diagnosis to 70%); and the proportion of undiagnosed HIV infection in the PWID population (from 39% for males and 22% for females, to 14% for each). Theses analyses were selected to challenge the sensitivity of our results to assumptions that are either difficult to confirm (see *Agent Behavior* in Additional file 1), or in the cases of diagnosis and viral suppression levels, due to competing methodologies [25, 35].

## Results

**Fig. 1:**
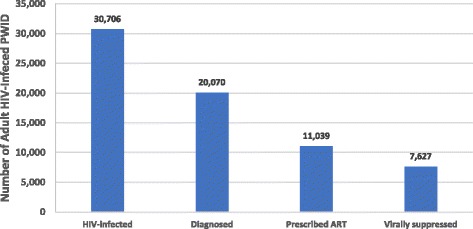
Estimated HIV Care Continuum for People Who Inject Drugs in New York City in 2012. Sources: [20, 23, 25]. Note: The number of HIV-infected people who inject drugs was estimated using data on the proportion of undiagnosed cases among men and women from the NHBS-IDU-3 New York City data, whereas the other values were estimated using surveillance data from the New York City Department of Health and Mental Hygiene. These estimates are only among adults, defined as aged >19 years. Column headings are defined as follows: HIV Infected – total persons with HIV infection; Diagnosed – total persons with diagnosed HIV infection; Prescribed ART – total persons prescribed ART for HIV infection; Virally suppressed – total persons who have achieved HIV viral suppression (viral load <200/mL)

The total estimated PWID in NYC living with HIV infection in 2012 are presented in Fig. 1, along with the estimated number of PWID who had received an HIV diagnosis, were receiving treatment, and were virally suppressed in 2012. The gaps between the bars in the figure (i.e., their numerical difference), along with the final estimate for those virally suppressed, represent the populations within the HIV care continuum categories used for this analysis (Undiagnosed, Diagnosed − not on ART, Unsuppressed, Suppressed).

Estimated HIV incidence among PWID in the model was 113 per 100,000 person-years over the study period, whereas the incidence rate for all adult New York City inhabitants was 33 per 100,000 person-years. These results are similar to our empirical estimates calculated using available observational data (see *Estimating HIV Prevalence and Incidence* in Additional file 1), where we estimated the HIV incidence rates for PWID and the overall adult population to be 107 and 32 per 100,000 person-years, respectively. The mean number of transmission events arising from HIV-infected PWID was approximately 378 per 100,000 person-years; of these transmissions, 52% were via parenteral transmission and 48% were transmitted sexually.

**Fig. 2:**
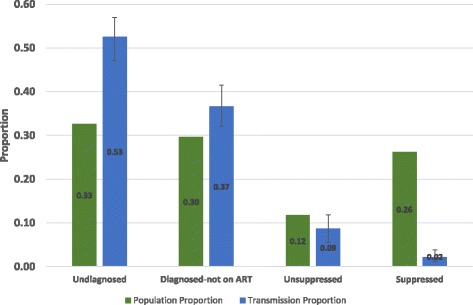
Population Size and Transmission Attributable to HIV Care Continuum Steps Among People Who Inject Drugs. Abbreviations: ART: antiretroviral therapy; HIV: human immunodeficiency virus. Note: The green bars (population proportion) correspond to the proportion of HIV-infected people who inject drugs in New York City in 2012 in a given step of the HIV care continuum. The blue bars (transmission proportion) correspond to the proportion of HIV transmission attributable to those in a given step of the HIV care continuum. The HIV care continuum categories are defined as follows: Undiagnosed - those without a positive HIV diagnosis; Diagnosed-not on ART - those positively diagnosed but not enrolled on ART; Unsuppressed - those enrolled on ART but not virally suppressed; Suppressed - those who have achieved viral suppression. The population proportion refers to the average proportion of HV-infected people who inject drugs in the respective care continuum step over the one-year study period. The error bars represent the 95% simulation interval obtained from the 10,000 Monte Carlo runs used to estimate the main results

The main results for HIV transmission within each step of the HIV care continuum are presented in Fig. 2. As shown, 33% of PWID in 2012 were undiagnosed, 30% were diagnosed but not on ART, 12% were prescribed ART but not virally suppressed, and 26% were prescribed ART and virally suppressed. For 2012, we estimated that 52.6% (95% simulation interval [95% SI]: 47.1–57.0%) of all HIV transmission events arising from PWID were from those who were undiagnosed, 36.6% (95% SI: 32.2–41.5%) from those who were diagnosed but who had not initiated ART, 8.7% (95% SI: 5.6–11.8%) from those who had been prescribed ART but failed to achieve viral suppression, and 2.1% (95% SI: 1.1–3.9%) from those enrolled on ART who have achieved viral suppression.

The undiagnosed PWID had the greatest ratio of transmission to population size, 1.61, followed by ratios of 1.23 for those Diagnosed-not on ART, 0.74 for Unsuppressed, and finally 0.08 for Suppressed.

**Fig. 3:**
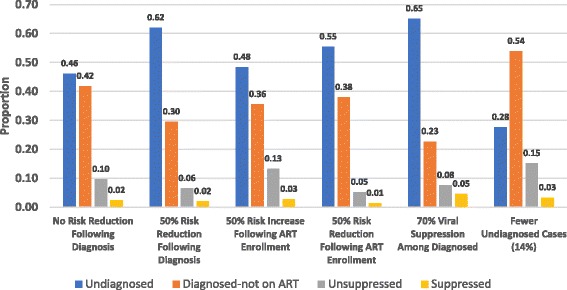
Transmission Attributable to HIV Care Continuum Steps Among People Who Inject Drugs: Sensitivity Analyses. Abbreviations: ART: antiretroviral therapy; HIV: human immunodeficiency virus. Note: The estimates presented here correspond to the proportion of HIV transmission attributable to those in a given step of the HIV care continuum among people who inject drugs in New York City in 2012. Each of the six panels on the horizontal axis present the results of sensitivity analyses wherein a specific assumption model assumption was adjusted from the main analysis. The HIV care continuum categories are defined as follows: Undiagnosed - those without a positive HIV diagnosis; Diagnosed-not on ART - those positively diagnosed but not enrolled on ART; Unsuppressed - those enrolled on ART but not virally suppressed; Suppressed - those who have achieved viral suppression

Results of the sensitivity analyses are presented in their respective panels in Fig. 3. As shown, the largest changes in results from the main analysis were observed when assuming fewer undiagnosed cases, or assuming a large reduction in risk behavior following HIV diagnosis. These differences are largely manifested by shifting estimated transmission to and from those with undiagnosed infection. The remaining analyses differed less substantially from those of the main analysis. Supplementary output (Additional file 1: Figures. S1 and S2) are available in the Additional file 1, presenting the proportion of HIV-infected PWID that had received an HIV diagnosis over the simulation period, as well as the proportion of all PWID diagnosed with HIV that were enrolled on ART, respectively. The original model ouputs used to generate the results presented here are available in Additional file 2, Additional file 3, Additional file 4, Additional file 5 and Additional file 6.

## Discussion

Using an agent-based modeling approach, we estimated that approximately one-third of all HIV transmission events arising from PWID occurs among those who have been diagnosed, yet have not begun ART, with an additional one-half occurring among those who have yet to be tested. Together, these steps accounted for an estimated 89% of all HIV transmission from PWID in 2012, despite only comprising 62% of the total infected population. These results are believed to be the first to examine PWID transmission along the HIV care continuum at a city-wide level, and indicate the importance of advancing PWID further along the care continuum, increasing rates of HIV serostatus awareness, ART initiation and viral suppression.

Although we estimated two-thirds of HIV-infected PWID to have diagnosed infection throughout 2012 in NYC, the magnitude the undiagnosed PWID population may have had on transmission was far greater than its relative size. As expected, out of the four points in the care continuum examined in this study, undiagnosed PWID produced the largest ratio of transmission to population size, at about 1.6. This result suggests that early diagnosis of HIV infection among PWID may provide the greatest opportunity to reduce incident infections on a per-person basis. Although we assumed reduced risk behavior following an HIV diagnosis (which is likely one reason for this result), there are other factors that may have influenced the likelihood of transmission from diagnosed compared to undiagnosed agents. For instance, the probability of being acutely-infected is higher among those with undiagnosed HIV infection (who are more likely to be recently infected), thus increasing transmission risk in this sub-group.

It should be noted that we assumed a non-zero probability of transmission for those with viral suppression, accounting for the transmission arising from this population. Although some equate viral suppression with an inability to transmit, it is unclear if or at what level of viral suppression this becomes absolute for an efficient mode of transmission such as parenteral exposure [36, 37]. Previous modeling work has similarly assumed a small probability of transmission, even from virally suppressed individuals [13].

From a national perspective, even though previous work has estimated that as many as 93% of all HIV-infected PWID may be previously diagnosed, only about 1 in 3 HIV-infected PWID was found to be virologically suppressed [38]. Delayed engagement in care and ART initiation have been found to be highly common among PWID [39, 40]. Research has shown several barriers to engagement in care, retention on treatment, and ART adherence, such as poverty, poor access to medical care, homelessness, and incarceration [39, 41, 42]. Despite substantial barriers to enrollment and retention, PWID often maintain a level of viral suppression (for those actively engaged in ART care) comparable to other high-risk groups in the U.S. [36]. This suggests that although treatment enrollment and retention may be hindered by significant structural and systemic barriers, the benefits of treatment initiation are high—as demonstrated by the comparably minor transmission shown in our results to originate from PWID on treatment.

Our results for NYC differed notably from earlier work by Skarbinsky, et al., who used a compartmental model to estimate HIV transmission along the care continuum for the entire U.S. [13]. We found a substantially higher contribution from PWID with undiagnosed infection (52.6% vs. 16.3%), but this difference was largely anticipated due to the stark differences between the estimated proportion of undiagnosed HIV infection among PWID in NYC (using the NHBS) and assumptions regarding the country as a whole made by Skarbinsky et al.

There are several limitations to our approach. The most significant of these is the challenge in correctly estimating the number of undiagnosed infections in NYC, among PWID, and other subpopulations. Some of our estimates for HIV diagnosis are based on a small sample size (particularly among female PWID). Additionally, specifying the behavior of diverse communities of PWID is inherently difficult (and at times based on older data), particularly considering the large range in potential sexual and injection risk behavior and network mixing [32]. In addition to the difficulty inherent in correctly specifying the HIV risk of diverse populations, it is possible that complex and confounding relationships exist between certain risk behaviors and different stages of the HIV care continuum (e.g., those less likely to engage in needle-sharing might also be more likely to be on HIV treatment and achieve viral suppression). Complex relationships such as this were not incorporated into our analyses. Future analyses may benefit from increased specification of the relationship between likelihood of engaging in risk behavior and stages along the HIV care continuum.

Methods for determining the likely route of HIV transmission among newly diagnosed cases are well documented [18–20]; however, their accuracy is not well understood, which makes it difficult to compare our results regarding the proportion of transmission attributable to sexual or parenteral exposures. This issue is inherently important among PWID, who have such disparate risk behaviors. Although our model calibration process largely dictated the proportion of transmission via a given route, empirical estimates among PWID are difficult to ascertain [43], and this would not have altered the total estimated incidence for the analysis, which was based on NYC DOHMH surveillance among all of those with reported IDU. The NYC DOHMH classifies any new infections among individuals reporting a history of IDU under either the IDU or MSM/IDU transmission categories. Both reporting bias and inclusion of individuals who were not actively injecting during the time of their HIV acquisition may have affected our results. Since our model only classifies those currently engaging in IDU as PWID, differences in classification by the NYC DOHMH and our model may have led to inaccurate estimates for incidence among PWID.

Although newer surveillance estimates suggest that the number of HIV-infected PWID in NYC may be overestimated [35], it is unclear what impact these results may have on the proportion of undiagnosed cases among PWID and others, even if levels of treatment engagement and viral suppression may be higher than previously estimated. Our analysis suggests that higher levels of virological suppression (i.e., 70% vs. 38%) substantially shifts estimates of transmission towards the undiagnosed population, but this assumes the size of the undiagnosed population remains as it was in the main analysis.

Certain model assumptions may have also influenced the results in potentially meaningful ways. For example, HIV testing was presumed to be 100% accurate and with no window period, leaving the possibility that some infections in the model were diagnosed earlier than they may have otherwise been with older generation testing. Finally, ART is presumed to have an immediate effect on suppression of viral load and disease progression, meaning that individuals on ART experience the benefits of full or partial adherence immediately, potentially overestimating the preventive effect of ART, which may take several weeks or months to become established [44]. Other modeling or observational approaches may be feasible to confirm our findings; however, observational approaches would require a substantially enhanced surveillance system, with robust estimates for risk behavior, viral sequencing, and treatment outcomes among PWID. Although more parsimonious models may be able to avoid some of the limitations of complex agent-based modeling, they may have difficulty reproducing the many advantages of this approach, including the reproduction of the sexual and injection risk network among PWID and with non-PWID partners.

## Conclusions

Among PWID in NYC in 2012, an estimated 89% of new HIV infections originate from persons who are not on ART. Our results demonstrate the importance of early diagnosis, as well as interventions to engage previously diagnosed PWID in care and treatment to further suppress population-level HIV transmission. With an estimated 53% of infections arising from undiagnosed PWID, further efforts should also be made to expand frequent HIV testing, which has been shown in prior research to be effective at forestalling transmission [45], but may also decrease transmission via reduced risk behavior post-diagnosis [46–48]. Future HIV prevention research should focus on the elimination of identified and potential barriers to the testing, diagnosis, and retention of PWID on HIV treatment.

## Additional files

Additional file 1: The Supplemental Material document contains detailed information on the agent-based model structure, calibration, and supplemental results. (DOCX 64 kb)

Additional file 2: Incidence Results. This file contains original model output for all simulation runs on the estimated number of incident HIV infections among specific subpopulations for each month for a scaled adult NYC population of 250,000 in 2012. (TXT 1457 kb)

Additional file 3: Population Results. This file contains original model output for all simulation runs on the estimated number of total HIV-infected persons, persons with diagnosed HIV infection, and persons enrolled on HAART for each month for a scaled adult NYC population of 250,000 in 2012. (TXT 4018 kb)

Additional file 4: PWID Results. This file contains original model output for all simulation runs on the estimated number of PWID in a given step of the HIV care continuum for each month for a scaled adult NYC population of 250,000 in 2012. (TXT 2729 kb)

Additional file 5: Transmission Results. This file contains original model output for batches of 100 simulation runs on the estimated number of HIV transmission events arising from a specific subpopulation for each month for a scaled adult NYC population of 250,000 in 2012. (TXT 75 kb)

Additional file 6: Transmission Type Results. This file contains original model output for all simulation runs on the estimated number of sexual and parenteral transmission events arising from PWID for each month for a scaled adult NYC population of 250,000 in 2012. (TXT 90 kb)

## Abbreviations

ART: Antiretroviral therapy
CDC: Centers for Disease Control and Prevention
DOHMH: Department of Health and Mental Hygiene
IDU: Injection drug use
MSM: Men who have sex with men
NHBS: National HIV Behavioral Surveillance
NYC: New York City
PWID: People who inject drugs
TasP: Treatment as Prevention
U.S.: United States
