# Supplementary material for: The risk of HIV transmission at each step of the HIV care continuum among people who inject drugs: a modeling study
Source: BMC Public Health. 2017 Jul 25;17:614. doi: 10.1186/s12889-017-4528-9 (PMC5525346; doi:10.1186/s12889-017-4528-9)
Supplement: Supplementary file 1 — The Supplemental Material document contains detailed information on the agent-based model structure, calibration, and supplemental results. (DOCX 64 kb) [file 12889_2017_4528_MOESM1_ESM.docx]

**Supplemental Material for:**

THE RISK OF HIV TRANSMISSION AT EACH STEP OF THE HIV CARE CONTINUUM AMONG PEOPLE WHO INJECT DRUGS: A MODELING STUDY

Daniel J Escudero^1^, Mark N Lurie, PhD^2^, Kenneth H Mayer, MD^3,4^, Maximilian King^2^, Sandro Galea, MD^5^, DrPH, Samuel R Friedman, PhD^6^, Brandon DL Marshall, PhD^2^

1. Department of Epidemiology, Harvard T.H. Chan School of Public Health, Boston, MA, USA
2. Department of Epidemiology, Brown University School of Public Health, Providence, RI, USA
3. Fenway Health, Boston, MA, USA
4. Beth Israel Deaconess Medical Center, Boston, MA, USA
5. Boston University School of Public Health, Boston, MA, USA
6. National Development and Research Institutes, New York, NY, USA

**This Supplemental Material includes additional information regarding the structure, calibration, and supplemental results for the agent-based model.**

Revised: 7/19/17

Number of Supplementary Tables/Figures: 5

Word Count: 5,864

**Contents**

1. **Study Objective p. 3**
2. **Data Sources and Baseline Parameter Values p. 4**
3. **Estimating HIV Prevalence and Incidence p. 5**
4. **Agent Population p. 9**
5. **Network Structure p. 10**
6. **Agent Behavior p. 12**
7. **HIV Disease Progression and Treatment p. 14**
8. **HIV Transmission p. 14**
9. **Model Calibration p. 18**
10. **Sensitivity Analyses p. 21**
11. **Technical Details p. 22**
12. **Supplementary Output p. 23**
13. **References p. 26**

**Study Objective**

The agent-based model (ABM) was developed to examine the HIV care continuum for people who inject drugs (PWID) in New York City (NYC) in 2012. Specifically, the model estimates the proportion of HIV transmission events arising from PWID for each identified stage in the care continuum. The care continuum stages as defined within the manuscript are: (**1**) those without a positive diagnosis, (**2**) those positively diagnosed but not enrolled on antiretroviral therapy (ART), (**3**) those prescribed ART but not virally suppressed, and (**4**) those who have achieved viral suppression (defined as a viral load <200 copies/mL).

Briefly, our results may help determine the prioritization of prevention resources for PWID along the HIV care continuum in NYC and similar settings, including potential strategies aimed at providing support to HIV-infected PWID who have not yet achieved viral suppression. Increasing the proportion of HIV-infected PWID who achieve viral suppression is critical in experiencing population-level reductions in incidence through the principle of treatment as prevention (TasP) [1-5]. Given the demonstrated risk for sexual transmission of HIV among PWID, this model is designed to estimate not only transmission via injection-drug use (IDU), but also through sexual activity, both between two PWID, and also from PWID to other members of the population. Therefore, our ABM is a population-based model that examines the total contribution of transmission from HIV-infected PWID.

Our model is adapted from previous versions of the ABM [6-8], which have investigated the roles of combination prevention strategies and treatment programs, as well as HIV transmission during acute infection on the epidemic (i.e., incidence) among PWID within NYC and the New York Metropolitan Statistical Area.

**Data Sources and Baseline Parameter Values**

The primary data sources for constructing the HIV care continuum for PWID in NYC were the HIV Health and Human Services Planning Council of the NYC Department of Health and Mental Hygiene (DOHMH) [9], and data collected in NYC as part of the IDU-3 sample of the National HIV Behavioral Surveillance (NHBS) system [10]. Full details on the NHBS, including published reports, are publicly available on the Center for Disease Control and Prevention’s (CDC) website: [www.cdc.gov/hiv/statistics/systems/nhbs/](http://www.cdc.gov/hiv/statistics/systems/nhbs/). Briefly, the NHBS is a surveillance system conducted in rotating, annual cycles among three populations at high risk for HIV: (1) gay, bisexual and other men who have sex with men; (2) PWID; and (3) heterosexuals at increased risk for HIV infection (e.g., partners of those who are HIV infection, those who engage in sex work) [10-12]. For PWID, the NHBS employs a respondent-driven sampling (RDS) technique, wherein a small number of initial “seeds” are selected to complete the study survey and in turn recruit peers (up to 5 each) to participate. This process continues until the target sample size of 500 participants is reached.

We used these data to estimate the proportion of PWID in each of the four HIV care continuum steps included in our analysis. As the manuscript describes, given previous results that have indicated PWID occupying the third step (retained in care but not prescribed ART) have a minimal effect on HIV transmission [13], and current efforts emphasizing the immediate treatment of all HIV-infected persons [14, 15], we decided to collapse the second and third steps into a single second step—as they are described in the *Study Objective* (Undiagnosed, Un-enrolled, Unsuppressed, and Suppressed).

Important model data sources are described in the manuscript in the *Model Data Sources* section. Most sources used to estimate baseline model parameters (i.e., demographics, risk behavior, transmission, partner formation) can be found in **Tables S1-S2**, as well as the sections below. As described in the *Model Calibration* section, some baseline parameters are modified based on the results of preliminary model runs (the results of which are compared to empirical estimates for HIV incidence and prevalence in key subpopulations within the model). The final values for parameters that were altered during calibration are presented in the *Model Calibration* section.

**Estimating HIV Prevalence and Incidence**

HIV prevalence among each subpopulation was calculated directly from surveillance data and estimates of the adult NYC population and subpopulation sizes. First, we obtained the total estimated number of HIV infected residents of NYC, as reported by the NYC DOHMH to be 131,766 in 2011 [9]. We then subtracted the estimated number of infections among those 19 years of age and younger, using the number of diagnosed infections among this age group (1,305) [16] and assuming that 14% of the entire HIV positive under-20 population has received a diagnosis—the overall assumed proportion of diagnosis among NYC residents [9, 17]—resulting in a final estimate of 1,517, that was subtracted from the total HIV positive population estimate.

With an estimate of 130,249 HIV positive adults >19 years, we calculated the estimated overall adult HIV prevalence to be 2.05%, assuming an adult population of 6,365,903 [18]. However, evidence from the NHBS-IDU-3 data collected in NYC suggested that the NYC DOHMH may have underestimated total HIV infections among PWID. The NHBS-IDU-3 data within NYC found that only 61.4% of male PWID and 77.8% of female PWID in NYC had received an HIV diagnosis [10], significantly less than the 86% assumed for all subpopulations in the NYC DOHMH estimate for 2011 [17]. Although these estimates result in an underlying assumption of a greater number of undiagnosed HIV infections among PWID in NYC than was assumed by the NYC DOHMH, these NYC-based data are very close to national estimates for male diagnoses (61.4% in NYC vs. 62.2% nationally), and actually presume fewer undiagnosed infections for females (77.8% in NYC vs. 68.0% nationally) [10].

In order to estimate the HIV prevalence within each subpopulation, we first obtained data from the NYC DOHMH’s online Epiquery resource, which provided estimates for the total number and proportion of all HIV-diagnosed persons within a given subpopulation [16]. We excluded those who were believed to have acquired infection through vertical transmission or the category denoted “Other.” We allocated the substantial portion of cases with “Unknown” risk factors (24.4% of all diagnosed cases) proportionally (i.e., based on their relative estimated share of the HIV positive population) among MSM, heterosexual males, and females. We did not allocate any of these “Unknown” cases to the PWID subpopulation, assuming that history of injection drug use would limit the probability of an individual being placed in the “Unknown” transmission category.

Given the number of HIV positive persons in each subpopulation within NYC, we then used empirical estimates of the total subpopulation sizes in NYC [19], to directly calculate the HIV prevalence in the respective subpopulation. The prevalence estimates for each subpopulation are presented in **Table S1**. Although all PWID are also members of one of the other three subpopulations, the majority of their characteristics are distinct from non-PWID within the same MSM, heterosexual male, or female subpopulation. For instance, a heterosexual male PWID will have a probability of HIV infection corresponding to the HIV prevalence estimate specifically for male PWID, rather than for the heterosexual male subpopulation.

For PWID, using data from EpiQuery, we assumed there to be 20,070 cases of HIV among PWID in NYC at the end of 2011 (14,252 among males and 5,818 among females). Using the diagnosis proportion estimates from NHBS-IDU-3 from NYC, we assumed there to be 30,706 HIV-infected PWID in NYC (23,225 among males, and 7,480 among females). We then used the HIV prevalence estimates from the NYC NHBS-IDU-3 report to estimate the total number of male and female PWID [20]. This enabled us to estimate the total prevalence of HIV among PWID in NYC (approximately 18.0%).

HIV incidence among PWID and within the entire NYC adult population for 2012 was estimated using surveillance data reported by the NYC DOHMH for 2012 (some of which was subsequently revised and published in the 2013 report), and in other cases supplemented via correspondence with NYC DOHMH staff [17, 21, 22]. Using these data, we estimated the total HIV incidence among persons >19 years in NYC in 2012 to be 2105 cases, with 152 among PWID. These estimates yielded an incidence rate of approximately 32 per 100,000 person-years among the general adult population and 107 per 100,000 person-years among PWID in 2012.

**Table S1: Demographic and Risk Characteristics for Simulated Adult New York City Population in 2012**

| **Variable** | **Population** | | | |  |  |  |  |
| --- | --- | --- | --- | --- | --- | --- | --- | --- |
|  | **MSM** | **HM** | **Female** | **PWID*** | **Source** |  |  |  |
| **Demographics** |  |  |  |  |  |  |  |  |
| Population size (% of total agent population) | 5.0 | 45.0 | 50.0 | 2.67 | Calculated, [19, 20] |  |  |  |
| HIV prevalence (%) | 22.0 | 0.03 | 0.90 | 17.0 (M), 22.3 (F) | Calculated, [16, 20] |  |  |  |
| AIDS prevalence (%) | 1.5 | | | | [23] |  |  |  |
| All-Cause Mortality Rate (per 1,000 person-years) |  | | | |  |  |  | [24] |
| Among HIV-uninfected agents | 7.2 | | | 20.6 | [25-27] |  |  |  |
| Among HIV-infected agents, not on ART | 18.5 | | | 32.1 | [28] |  |  |  |
| Among HIV-infected agents, on ART | 7.2 | | | 20.6 | [25-27] |  |  |  |
| Among agents diagnosed with AIDS | 76.0 | | | 81.9 | [23, 25] |  |  |  |
| **HIV Risk Behaviors** |  | | | |  |  |  |  |
| Mean monthly sex acts with primary partner** | 2.8 | 3.4 | 4.6 | - | [29, 30] |  |  |  |
| Mean number of needle-sharing acts (per month/per partner) | - | | | 3.5 | Calculated, [31, 32] |  |  |  |
| Fraction of sex acts unprotected** | 0.58 | 0.77 | 0.67 | 0.25 | [20, 33, 34] |  |  |  |
| Reduction in risk behavior following HIV diagnosis (%) | 50 | | | 25 | [11, 35-38] |  |  |  |
| Proportion engaging in receptive needle sharing (per act) | - | | | 0.025 | Assumed/Calibrated |  |  |  |
| **Network Parameters** |  | | | |  |  |  |  |
| Mean number of partners | 2.3 | 1.5 | 1.2 | 3.8 (M), 6.7 (F) | [6, 20, 39] |  |  |  |
| Assortative Mixing (%)*** | 95 | 100 | | 80 | [31, 40] |  |  |  |
| **HIV Testing** |  |  | |  |  |  |  |  |
| Proportion obtaining an HIV test (annually)** | 0.22 | 0.09 | 0.16 | 0.22 | [12, 19, 20, 34] |  |  |  |
| **HIV Treatment Parameters** |  |  |  |  |  |  |  |  |
| Proportion diagnosed at baseline | 0.77 | 0.86 | 0.86 | 0.61 (M), 0.78 (F) | [9, 10, 12, 41] |  |  |  |
| Proportion of those diagnosed on ART at baseline | 0.57 | 0.63 | 0.63 | 0.55 | [9] |  |  |  |
| Proportion achieving viral suppression**** | 0.84 | 0.76 | 0.76 | 0.69 | [9] |  |  |  |

Abbreviations: AIDS: acquired immune deficiency syndrome; ART: antiretroviral therapy; HIV: human immunodeficiency virus; PWID: people who inject drugs

* Where estimates differ among male and female PWID, they are indicated by an “M” and “F”, respectively

** Baseline values were subject to alteration based on model calibration, final parameter values can be found in the *Model Calibration* Section

*** Defined as proportion of partners that originate from the same sexual preference or injection-drug use strata

**** Viral suppression is defined as having a viral load measurement of <200 copies/mL

Note: HIV prevalence within each subpopulation as well as the overall PWID population size were calculated using surveillance estimates obtained from the New York City Department of Health and Mental Hygiene and estimates of population size, as described in the Estimating HIV Prevalence and Incidence section. The mean number of needle-sharing acts per month/per partner were calculated based on the data from empirical sources. The proportion engaging in receptive needle sharing for a given injection act was calibrated downward starting from the reported proportion of PWID in NYC engaging in receptive needle sharing over a year.

**Agent Population**

The ABM consists of individual agents (or ‘nodes’) that represent individuals within a virtual population. Agents are stratified in three fundamental ways: IDU status (PWID vs. non-PWID); sex (male vs. female); and if the agent is a male, by sexual preference (heterosexual male vs. male who has sex with men [MSM]). Given minimal transmission via sexual activity among women who have sex with women [23], all females in the ABM are presumed to be heterosexual.

PWID agents are the only individuals in the model capable of engaging in IDU; however, agents may initiate or discontinue IDU based on empirical estimates [7, 42]. Heterosexual agents only engage in sexual relationships with those of the opposite sex. In contrast, some MSM are able to form sexual relationships with either sex, while most MSM form relationships exclusively with other MSM. For the purposes of this analysis, sex and sexual preference assignment for each agent is presumed to be time invariant. Agents may be characterized by any non-mutually exclusive set of the fundamental strata (i.e., IDU status, sex, sexual preference); for instance, an agent may be an MSM-PWID, or a female non-PWID. For PWID agents, their agent-specific parameters always supersede those of the other sex- and sexual preference-specific parameters where there is non-congruence. For example, the monthly mortality estimates for PWID agents are much higher than for all other agent classes, and thus a heterosexual male PWID will be assigned the PWID value for mortality rather than the value for heterosexual males. Similarly, PWID agents, particularly female PWID, have a higher mean number of sexual partnerships than non-PWID agents.

The probability of being assigned a given set of characteristics (i.e., sex, sexual preference, and IDU status) is determined by the prevalence of these populations as estimated from empirical data for the sexually-active adult U.S. population, where available [19, 43]. The distribution of these population characteristics is presented in **Table S1**. As described in the *Estimating HIV Prevalence and Incidence* section, our HIV prevalence calculations yielded specific estimates for the number of male and female PWID in NYC: 136,620 male PWID and 33,544 females PWID, creating a male to female PWID sex ratio of approximately 4:1.

This means that, in the baseline ABM, a male agent would have a roughly 4-fold chance of being classified as a PWID, and the overall probability of an agent being classified as a PWID is 2.67%. However, due to small probabilities of initiation or cessation of IDU for individual agents, based on empirical data [7, 42], over the course of the simulation period the precise distribution of PWID agents may vary (see *Agent Behavior*).

**Network Structure**

At model initialization, there are 250,000 agents within the virtual population, at which point they begin forming connections with other agents (i.e., via an ‘edge’) that represent one of three potential relationships: sexual, sexual and injecting, or injecting only. After initialization, the model moves forward through time in discrete time-steps that represent a month of elapsed time. During these transitions between time-steps, agents stochastically form, dissolve, or maintain their current relationship connections. For instance, if Agent **X** is connected at time-step *t* to one other agent (Agent **Y**), at time-step *t*+1, Agent **X** may form another concurrent relationship with one or more of the remaining agents in the model, dissolve the relationship with Agent **Y**, or maintain a monogamous relationship with Agent **Y**.

To construct the network (i.e., form relationships between agents), the program assigns a value $k_{i,t}$ to each index agent 𝑖, where $k_{i,t}$ is defined as the number of partnerships with other agents per time step 𝑡. The value $k_{i,t}$ is determined by a random sampling procedure from negative binomial (NB) distribution functions, i.e.:

**Equation 1**

$$K_{i,t}\sim NB\left( p,r \right)=\frac{\left( k_{i,t}+r-1 \right)!}{\left( r-1 \right)!k_{i,t}!}p^{r}\left( 1-p \right)^{k_{i,t}}, k_{i,t}\in\aleph_{0}$$

with mean given by:

**Equation 2**

$$m=\frac{pr}{1-p}$$

for all agents per time step. This method of partner formation means that partners are acquired with probability *p* until *r* suitable partners are found. A previous version of the ABM has used negative binomial distributions to determine partnership formation [6], and the use of NB distributions have been shown by other studies to provide reasonable approximations of real-world partnership networks, in which the variance of the distribution is greater than would be expected assuming constant-rate function (e.g., Poisson) [44].

We define NB distribution functions representing sexual relationships for each of the following mutually exclusive categories of non-PWID agents: MSM, heterosexual males, and females.

These functions are based on available data from surveys of sexual partnerships. The majority of these values are extrapolated from studies with published annual estimates, and applied to the ABM’s monthly time-steps (with a turnover probability function, see below, that reduces annual unique partnerships). The sources for these partner estimates are found in **Table S1**. For PWID agents, however, we were unable to assign NB distributions to fit the data found by NHBS-IDU-3 as reported by the NYC DOHMH [20]. These data suggested that the number of partnerships over a year for male PWID had a mean of 3.8 and median of 2.0, with females reporting a mean of 6.7 and a median of 2.0. Therefore, we designed distributions using a beta function to fit each of these specific estimates.

To avoid overestimating the number of unique partners over consecutive monthly sample drawings, we assigned a partner turnover function that assumes each agent experiences a potential partner turnover event (where they redraw from the partnership distribution) on average once a year. This method ensures that most agents will not form an unrealistic number of unique partnerships over a given time period, and increases the fidelity among partners. A monthly probability value of 0.08 was selected so that over the course of 12 months, each agent may expect to experience a turnover event approximately one time.

**Agent Behavior**

Agents who share a link in the network can engage in sexual and/or injecting behavior at each time-step. We based our assumptions of sexual and needle-sharing behavior on literature estimates [31, 40], which suggest that PWID frequently engage in both of these behaviors with the same partners, so our partner structure was designed to replicate this phenomenon. A PWID-PWID agent dyad, for example, can engage in sexual activity exclusively, injecting activity exclusively, or both, with probabilities 0.20, 0.60, and 0.20, respectively, based on published studies [31, 40].

The probability that a PWID agent engages in receptive needle sharing during any given injection act with a PWID partner is presented in **Table S1**; also shown are the probabilities that a given agent will engage in unprotected sex. If two connected agents have a different probability of engaging in unprotected sex, then the model automatically assigns the unprotected sex probability of the HIV positive agent within the dyad.

The baseline number of sexual or needle sharing acts in which a given dyad engage (within a given month) is determined stochastically using a Poisson-distributed estimate, with a mean presented for the corresponding agent and risk type in **Table S1**. In order to calculate the overall transmission risk per partnership per time step, $\beta_{p}$, we employed a Binomial process model [45], i.e.:

**Equation 3**

$$\beta_{p}\sim Bin\left( n,\beta_{a} \right)=\frac{\beta_{p}!}{\left( \beta_{p}-n \right)!n!}{\beta_{a}}^{\beta_{p}}\left( 1-\beta_{a} \right)^{n-\beta_{p}},\beta_{p}\epsilon\left\{ 1,\cdots,n \right\}$$

where $\beta_{a}$ is the per-act transmission probability, specific to the type of risk activity engaged in between two serodiscordant agents (i.e., parenteral, anal intercourse, or vaginal intercourse). The number of trials, *n*, is equal to the number of acts, as determined by the Poisson distribution described earlier.

Although sex and sexual preference are time invariant characteristics (i.e., they do not change), agents can initiate or cease drug use at any month. PWID have a 0.2% chance of spontaneous drug use cessation, based on previous research [42], at which point they join the non-PWID class of agents. The probability that an agent transitions to IDU (i.e., becomes a PWID) was determined in previous model analyses, inductively from a calibration procedure, that sought to reproduce empirical estimates of PWID prevalence [7]. These probabilities were transformed, as other previously calibrated parameters, to apply to monthly rather than annual time steps.

As noted in the manuscript, for the main analysis, we assumed that risk behavior remained constant following initiation of ART, as was found in several observational studies of ART [46-48]; however, the sensitivity of our results to this assumption were tested in sensitivity analyses. We also assumed that risk behavior for non-PWID agents decreased by 50% after HIV diagnosis, and 25% for PWID, based on empirical observations [35-37]; these, too, were subject to sensitivity analyses.

Although we did not explicitly model main and secondary partnership types (i.e., assign different partnership durations, probability of unprotected sex), the model assumes a significantly lower number of sex acts for partners randomly designated as non-primary partners. We assign a probability that determines each month whether a sex act will occur with a given secondary partner. Additionally, if agents do engage in sex with a secondary partner, this interaction has a mean of only 1 sex act per month, rather than the mean value for the primary partner, as presented in **Table S1**. Similarly, there is a probability that a PWID agent will engage in receptive needle-sharing with a partner for a given risk act. These values were determined during the calibration procedures (see *Model Calibration*).

We used data from the NHBS and elsewhere to estimate potential reductions in sexual or injection risk behavior following HIV diagnosis among PWID, MSM and the heterosexual populations within the model [35-38, 49]. Despite early research indicating that PWID may engage in increased risk behavior following enrollment on ART [50], more recent studies have suggested no change in either sexual or injection risk behavior among PWID who initiate ART [46, 47]. Subsequently, in primary analyses, we assumed no change in risk behavior following ART initiation. Given the importance of these assumptions for our primary results, these assumptions, among others, were subject to sensitivity analyses.

**HIV Disease Progression and Treatment**

A detailed description of our HIV disease progression model has been published previously [7]. Following acute HIV infection (AHI), which lasts for 3 monthly time steps, based on previous data [51], HIV positive agents in latent stage infection progress to AIDS at a rate dependent on treatment adherence. This approach assures that there will be a large variation in time-to-AIDS for HIV positive agent population, but also has a notable limitation in that all agents may progress to AIDS with equal probability at each point following AHI, meaning that a very small portion may progress to AIDS sooner than population-level estimate and clinical case-studies suggest [52]. However, these instances of early progression are very rare. The probability of progression to AIDS for each adherence category is listed in **Table S2**. There is a baseline probability of mortality for each agent class, as well as an increased probability of mortality for HIV-infected agents, based on their AIDS and ART statuses; these values are presented in **Table S1**. The portion of ART enrollees that achieve >90% adherence (assumed to be virally suppressed) was estimated using NYC DOHMH data [9]; however, the proportion of ART enrollees in each of the partially adherent categories (e.g., 0-29%, 30-49%, 50-69%, and 70-89%) was assumed to be equally distributed. Agents on ART may discontinue therapy at any time, based on previously published literature [53-57].

**HIV Transmission**

If an agent dyad consists of an HIV discordant pair (i.e., one agent is HIV-infected and one agent is HIV-uninfected), then HIV transmission is possible, through either sexual or injection risk behavior. During each time step in which the agents engage in risk behavior, the ABM stochastically determines whether a transmission event will occur.

To calculate the probability of HIV transmission to an uninfected partner for each type of risk behavior, we use per-act probabilities shown in **Table S2**, based on previously published estimates [58-65]. We do not explicitly model partner roles in sexual risk acts among MSM (i.e., receptive or insertive); rather, the HIV negative partner has a probability of infection based on the average of receptive and insertive transmission probabilities. Furthermore, we did not model unprotected anal intercourse between women and men. The probabilities listed in **Table S2** represent the average risk of transmission during an unprotected coital act or syringe-sharing event during latent stage HIV infection. Viral load is modeled implicitly, such that these values represent a mean set-point viral load (approximately 4 log_10_ copies/mL) in the chronic HIV-infected population [66].

**Table S2: HIV Transmission and Disease Progression Parameters**

| **Variable** | **Not on ART** | **Adherence to ART** | | | | | **Source** |
| --- | --- | --- | --- | --- | --- | --- | --- |
|  |  | **0 – 29%** | **30 – 49%** | **50 – 69%** | **70 – 89%** | **≥90%*** |  |
| **HIV Disease Progression Parameters** |  |  |  |  |  |  |  |
| Progression to AIDS (monthly probability)** | 0.0013 | 0.0013 | 0.0010 | 0.0008 | 0.0006 | 0.0002 | [67-69] |
| **HIV Transmission Parameters***** |  |  |  |  |  |  |  |
| Risk per syringe sharing act (chronic phase) | 0.0070 | 0.0070 | 0.0056 | 0.0028 | 0.0014 | 0.0002 | [58, 59, 70] |
| Risk per unprotected anal sex act (chronic phase) | 0.0050 | 0.0050 | 0.0040 | 0.0020 | 0.0010 | 0.0001 | [60, 61, 63] |
| Risk per unprotected vaginal sex act (chronic phase) | 0.0010 | 0.0010 | 0.0008 | 0.0004 | 0.0002 | 0.0001 | [60, 62, 64, 65] |

Abbreviations: AIDS: acquired immune deficiency syndrome; ART: antiretroviral therapy; HIV: human immunodeficiency virus

Note: Transmission parameters are based on a single sexual or injection risk act, however many partnerships experience more than one risk act per month, subsequently the probability of transmission may be seen as binomially distributed based on the total number of acts (see *Equation 3*)

*For the analysis, only those in the ≥90% adherence category were presumed to be virally suppressed.

**These values are the final estimated values for AIDS progression, which were adjusted (reduced) from the baseline estimates by a factor of 0.75 (see *Model Calibration*)

*** These values correspond to $\beta_{p}$ in *Equation 3*

The values of the transmission probabilities for AHI were presumed to be relative to the corresponding values for each agent and risk type. Agents with AHI were presumed to experience acute infection for three months. For our analysis, the best available approximations for relative infectiousness were used. These estimates (either explicitly calculated by other investigators, or our team using per-act viral load specific estimates) ranged between about 2-fold to 26-fold increased infectiousness of AHI to chronic infection [51, 63, 71, 72]. Subsequently, the model assumes a 10-fold relative infectiousness for AHI, approximately the same ‘middle range’ value, of 9.2 used in the main analysis for another recent study by Eaton et al. examining acute infection transmission [73]. ART enrollment and the corresponding adherence classes each attenuate the per-act probability of HIV transmission, and the resulting parameter estimates are listed in **Table S2**.

The manuscript provides an estimate for the rate of transmission from HIV-positive PWID, which was calculated using the mean number of transmission events arising from PWID in each model run (approximately 68 per 100,000 PWID PY), and the prevalence of HIV within the PWID population (18%). This yielded an approximation of 378 transmissions from HIV-positive PWID per 100,000 PY.

**Model Calibration**

To calibrate the model, we employed an iterative indirect approach following previously published recommendations [74]. First, the set of empirical behavioral and risk parameters were applied to the model agents, and preliminary outputs (primarily HIV incidence) were assessed and compared to surveillance data from the NYC DOHMH [16, 17, 21, 22] or other empirical data where appropriate.

Model refinement was then conducted by adjusting key parameters for which there existed greater uncertainty in their values (e.g., the mean number of sexual/injection risk acts per month, the proportion of secondary partners with whom a given agent interacts with sexually each month, the proportion of injection acts that involve receptive needle sharing) to minimize differences between model output and empirical data. We lacked any reliable estimates for the proportion of injection acts that involved receptive syringe sharing, so we began the calibration process using an estimate for the proportion of NYC PWID who engaged in needle-sharing over a one year period (31%) [20], and gradually adjusted this value downward (along with mean injection acts) to approximate a per-act probability of needle-sharing. The mean number of sexual risk acts for each subpopulation, the probability of having unprotected sex for PWID, and the probability of sexually interacting with a secondary partner were also adjusted concurrently until HIV incidence among the general population and the PWID population approximated NYC DOHMH estimates. Although this process does not necessarily guarantee model validity, it does permit the exclusion of parameter values that do not adequately reproduce the empiric data [74].

To estimate the HIV incidence rate among the PWID subpopulation in the model, we divided the total number of incident cases among PWID over the 10,000 Monte Carlo runs by the average number of PWID in the agent population, 6,675 (2.67% x 250,000 total agents), who contribute about one year of follow-up within the study period. In preliminary results, the baseline parameter estimates described above produced incidence greater than estimated from the NYC DOHMH, and were therefore incrementally adjusted until incidence roughly matched empirical estimates.

In the final calibrated results, PWID have an incidence rate of 113 per 100,000 person-years, while the general population incidence rate was 33 per 100,000 person-years, approximately equal to extrapolations from NYC DOHMH estimates described earlier in the *Estimating HIV Incidence and Prevalence* section (107 and 33 per 100,000 person-years, respectively). In addition, the baseline probability of progression to AIDS substantially increased the proportion of HIV positive agents with AIDS over the course of the year, subsequently this value was reduced to create an approximately stable prevalence of AIDS within the HIV positive community.

One source of significant uncertainty in the model was the rate of HIV testing within each subpopulation. We began the analysis using past-year HIV testing estimates collected by the NHBS and other empirical studies [12, 19]. These self-reported data may have overestimated the true frequency of HIV testing among each subpopulation, and we substantially reduced these values during calibration (see **Table S3**) so that the number of new HIV diagnoses in the model approximated the number of new diagnoses reported by the NYC DOHMH (using 2012 values from the 2013 report, which contained revised estimates from the previous report) [22]. We subtracted 147 HIV diagnoses in 2012 that occurred among persons <19, yielding a target of 1,843 HIV diagnoses over the study period.

As a means of ensuring that HAART enrollment estimates among the general and PWID populations within the twelve-month study period approximated empirical observations, we compared the level of viral suppression during the final month of 2012 with NYC surveillance data from 2012. These data suggested that approximately 49% of diagnosed cases within the general population had achieved viral suppression by the end of 2012 [75], an increase from our baseline value of 47.3%. Without specific data on each subpopulation, we assumed that all subpopulations would experience approximately the same percentage increase in viral suppression among those diagnosed over the twelve-month period. The baseline estimates for new HAART enrollment per month as estimated during previous analyses using the ABM [8], resulted in HAART enrollment within all subpopulations to be out-paced by new diagnoses, and did not converge on the suggested levels of viral suppression (they remained approximately stable from baseline levels; subsequently these values were increased until they approximated the increase suggested by the data from the end of 2012 [75].

**Table S3** presents the baseline values for each of the parameters adjusted during calibration. Well documented and previously published calibrated outputs for other variables of interest (e.g., HIV disease progression rates, see **Tables S1 and S2**) remained unchanged [76, 77].

**Table S3. Comparison of Initial and Final Calibrated Values for Model Parameters**

| **Parameter** | **MSM** | **Heterosexual Men** | **Heterosexual Women** | **PWID** |
| --- | --- | --- | --- | --- |
| Initial mean sex acts with primary partner^$^ | 2.8 | 3.4 | 4.6 | N/A |
| Final mean sex acts with primary partner | 2.2 | 2.7 | 3.7 | N/A |
|  |  |  |  |  |
| Initial proportion of unprotected sex acts | - | - | - | 0.76 |
| Final proportion of unprotected sex acts | - | - | - | 0.25 |
|  |  |  |  |  |
| Initial proportion engaging in receptive needle sharing (per act) ^$^ | - | - | - | 0.31 |
| Final proportion engaging in receptive needle sharing (per act) | - | - | - | 0.02 |
|  |  |  |  |  |
| Initial Probability of Progression to AIDS | * | * | * | * |
| Final Probability of Progression to AIDS | * | * | * | * |
|  |  |  |  |  |
| Initial Proportion Obtaining an HIV test (annually) | 0.65 | 0.27 | 0.48 | 0.66 |
| Final Proportion Obtaining an HIV test (annually) | 0.22 | 0.09 | 0.16 | 0.22 |
|  |  |  |  |  |
| Initial Probability of HAART Enrollment (per month) | 0.01167 | 0.01167 | 0.01167 | 0.00345 |
| Final Probability of HAART Enrollment (per month) | 0.01000 | 0.01000 | 0.01000 | 0.00800 |

Abbreviations: HAART: highly active antiretroviral therapy; MSM: men who have sex with men; PWID: people who inject drugs

^$^This baseline value was estimated as the proportion of PWID that reported engaging in receptive needle sharing in the past year, and this value was subsequently reduced during calibration to approximate the corresponding monthly value, but was decreased further as well during calibrations for HIV incidence among PWID.

*See Table S2 for the probability of progression to AIDS stratified by HAART status and adherence category; all final probabilities presented in Table S2 are ¼ the value of the initial estimates.

Note: A dash signifies no changes was made from the baseline parameter value.

**Sensitivity Analyses**

As described in the manuscript, sensitivity analyses were performed on factors suspected to strongly influence the main results. Specifically, we conducted four series of one-way sensitivity analyses, where the following were adjusted: the reduction in agent risk behavior following a positive HIV test (from a reduction of 25% to reductions of 0% and 50%); the change in agent risk behavior following ART initiation (from no change to a 50% increase and a 50% reduction); the level of viral suppression (from approximately 44% of those with an HIV diagnosis to 70%); and the proportion of undiagnosed HIV infection in the PWID population (from 39% for males and 22% for females, to 14% for each). Theses analyses were selected to challenge the sensitivity of our results to assumptions that are either difficult to confirm, or in the cases of diagnosis and viral suppression levels, due to competing methodologies [9, 75]. For the viral suppression sensitivity analysis, since the published methods did not provide a category for those currently enrolled on ART, we assumed that all those in the “retained in continuous care” were enrolled on ART (since our methods required that we assign one of our four care continuum steps for each HIV positive agent). Correspondingly, we assumed that 81% of those with an HIV diagnosis were enrolled on ART, and 85% of those individuals had achieved viral suppression—resulting in approximately 70% viral suppression among those with an HIV diagnosis.

The results on PWID transmission for each stage of the care continuum for all sensitivity analyses are presented in **Figure 3** in the manuscript.

**Technical Details**

The model was coded, tested, and calibrated in an open-source programming language (Python™ version 2.7.2). The simulation generated an agent matrix of 250,000 agents of varying classifications and substrata, which were managed by independent Python dictionaries. At each time step, information on the current agent state and each agent’s partners were recorded, agents were assigned partners using the methods described above, and then interacted with each other along their network edges. All agents performed their acts simultaneously during a time step, requiring careful consideration to the order of operations and transition of states of each agent at this time.

The program was run on a Beowulf supercomputing cluster consisting of multiple compute nodes and one head node, each with quad-core Intel™ CPUs and at least 8 GB of RAM. The model was run for a duration of 12 time steps (1 year) and averaged over a total of 10,000 Monte Carlo runs, each with a stochastically generated population following the parameters provided in **Table S1**. These 10,000 runs were aggregated from bundles of 100-run units, with each unit having an average runtime of approximately 2-3 hours.

**Supplementary Output**

Important model results that were not made available in the manuscript are presented here. **Figures S1 and S2** present model outputs by their mean value (over 10,000 Monte Carlo runs) at each month with the 2012 study period. **Figure S1** presents the proportion of HIV-infected PWID that have received an HIV diagnosis over the simulation period; **Figure S2** presents the proportion of all PWID diagnosed with HIV that are currently enrolled on ART.

**Figure S1. Estimated Proportion of Adult HIV-Infected People Who Inject Drugs in New York City That Have Ever Received an HIV Diagnosis**

Abbreviations: ART: antiretroviral therapy; HIV: human immunodeficiency virus

Note: Results represent mean values from 10,000 Monte Carlo runs. Simulated months represent the period January – December, 2012

**Figure S2. Estimated Proportion of Adult HIV-Infected People Who Inject Drugs in New York City That Have Enrolled on ART Among Those with an HIV Diagnosis**

Abbreviations: ART: antiretroviral therapy; HIV: human immunodeficiency virus

Note: Results represent mean values from 10,000 Monte Carlo runs. Simulated months represent the period January – December, 2012

**References**

1. Tanser, F., et al., *High coverage of ART associated with decline in risk of HIV acquisition in rural KwaZulu-Natal, South Africa.* Science, 2013. **339**(6122): p. 966-71.

2. Wood, E., et al., *Longitudinal community plasma HIV-1 RNA concentrations and incidence of HIV-1 among injecting drug users: prospective cohort study.* BMJ, 2009. **338**: p. b1649.

3. Wood, E., M.J. Milloy, and J.S. Montaner, *HIV treatment as prevention among injection drug users.* Curr Opin HIV AIDS, 2012. **7**(2): p. 151-6.

4. Montaner, J.S., et al., *Association of highly active antiretroviral therapy coverage, population viral load, and yearly new HIV diagnoses in British Columbia, Canada: a population-based study.* Lancet, 2010. **376**(9740): p. 532-9.

5. Cohen, M.S., et al., *Prevention of HIV-1 infection with early antiretroviral therapy.* N Engl J Med, 2011. **365**(6): p. 493-505.

6. Marshall, B.D., et al., *Prevention and treatment produced large decreases in HIV incidence in a model of people who inject drugs.* Health Aff (Millwood), 2014. **33**(3): p. 401-9.

7. Marshall, B.D., et al., *A complex systems approach to evaluate HIV prevention in metropolitan areas: preliminary implications for combination intervention strategies.* PLoS One, 2012. **7**(9): p. e44833.

8. Escudero, D.J., et al., *Acute HIV infection transmission among people who inject drugs in a mature epidemic setting.* AIDS, 2016. **30**(16): p. 2537-2544.

9. HIV Health & Human Resources: Planning Council of New York. HIV care cascades for New York City overall and Ryan White clients: A first look. http://www.nyhiv.org/pdfs/NAC%20Presentation%206-13-13.pdf. Accessed June 7 2016

10. Centers for Disease Control and Prevention. HIV Infection, Risk, Prevention, and Testing Behaviors among Persons Who Inject Drugs—National HIV Behavioral Surveillance: Injection Drug Use, 20 U.S. Cities, 2012. HIV Surveillance Special Report 11. Revised edition. http://www.cdc.gov/hiv/ library/reports/surveillance/. Published August 2015. Accessed May 3 2016.

11. Centers for Disease Control and Prevention. HIV Infection, Risk, Prevention, and Testing Behaviors among Heterosexuals at Increased Risk of HIV Infection—National HIV Behavioral Surveillance, 20 U.S. Cities, 2013. HIV Surveillance Special Report 13. http://www.cdc.gov/hiv/library/reports/surveillance/#panel2. Published August 2015. Accessed May 3 2016.

12. Centers for Disease Control and Prevention. HIV Risk, Prevention, and Testing Behaviors— National HIV Behavioral Surveillance System: Men Who Have Sex With Men, 20 U.S. Cities, 2011. HIV Surveillance Special Report 8. http://www.cdc.gov/hiv/library/reports/surveillance/-special. Published September 2014. Accessed May 5 2016.

13. Skarbinski, J., et al., *Human immunodeficiency virus transmission at each step of the care continuum in the United States.* JAMA Intern Med, 2015. **175**(4): p. 588-96.

14. Ying, R., R.V. Barnabas, and B.G. Williams, *Modeling the implementation of universal coverage for HIV treatment as prevention and its impact on the HIV epidemic.* Curr HIV/AIDS Rep, 2014. **11**(4): p. 459-67.

15. Camlin, C.S., et al., *Strengthening universal HIV 'test-and-treat' approaches with social science research.* AIDS, 2016. **30**(6): p. 969-70.

16. The City of New York. HIV/AIDS Surveillance Data: New York City (“EpiQuery”). https://a816-healthpsi.nyc.gov/epiquery/. Accessed December 9 2016

17. Personal Communication. Lisa Forgione, New York City Department of Health and Mental Hygeine. Tuesday, September 20, 2016 at 9:20 AM.

18. New York City Department of Health and Mental Hygiene. New York City HIV/AIDS Annual Surveillance Statistics. http://www.nyc.gov/html/doh/html/data/hivtables.shtml. Accessed June 6 2016

19. Pathela, P., et al., *Men who have sex with men have a 140-fold higher risk for newly diagnosed HIV and syphilis compared with heterosexual men in New York City.* J Acquir Immune Defic Syndr, 2011. **58**(4): p. 408-16.

20. The City of New York. HIV Risk and Prevalence among New York City Injection Drug Users: 2012 National HIV Behavioral Surveillance Study. http://www1.nyc.gov/assets/doh/downloads/pdf/dires/nhbsidu3-ppg-6-2013.pdf. Accessed December 9 2016

21. HIV Epidemiology and Field Services Program. HIV Surveillance Annual Report, 2012. New York City Department of Health and Mental Hygiene: New York, NY. December 2013.

22. HIV Epidemiology and Field Services Program. HIV Surveillance Annual Report, 2013. New York City Department of Health and Mental Hygiene: New York, NY. December 2014.

23. Centers for Disease Control and Prevention. HIV Surveillance Report, 2014; vol. 26. http://www.cdc.gov/hiv/library/reports/surveillance/. Published November 2015. Accessed June 6 2016.

24. U.S. Census Bureau. *QuickFacts: Atlanta (city), Georgia*. 2013. http://quickfacts.census.gov/qfd/states/13/1304000.html. Accessed January 6 2016

25. Mathers, B.M., et al., *Mortality among people who inject drugs: a systematic review and meta-analysis.* Bull World Health Organ, 2013. **91**(2): p. 102-23.

26. State Health Facts: Number of Deaths per 100,000 Population. Kaiser Family Foundation. http://kff.org/other/state-indicator/death-rate-per-100000/. Accessed Feb 2 2016

27. CDC Wonder. Centers for Disease Control and Prevention. http://wonder.cdc.gov. Accessed Feb 2 2016

28. Siddiqi, A.E., et al., *Mortality among blacks or African Americans with HIV infection--United States, 2008-2012.* MMWR Morb Mortal Wkly Rep, 2015. **64**(4): p. 81-6.

29. Leichliter, J.S., et al., *The concentration of sexual behaviours in the USA: a closer examination of subpopulations.* Sex Transm Infect, 2010. **86 Suppl 3**: p. iii45-51.

30. Wall, K.M., R. Stephenson, and P.S. Sullivan, *Frequency of sexual activity with most recent male partner among young, Internet-using men who have sex with men in the United States.* J Homosex, 2013. **60**(10): p. 1520-38.

31. Kottiri, B.J., et al., *Risk networks and racial/ethnic differences in the prevalence of HIV infection among injection drug users.* J Acquir Immune Defic Syndr, 2002. **30**(1): p. 95-104.

32. Friedman, S.R., Curtis, R., Neaigus, A., Jose, B., Des Jarlais, *Social Networks, Drug Injectors' Lives, and HIV/AIDS*. 1999, New York, NY: Kluwer Academic.

33. Haben, C.M., *Voice rest and phonotrauma in singers.* Med Probl Perform Art, 2012. **27**(3): p. 165-8.

34. The City of New York. HIV Risk and Prevalence among New York City High-Risk Heterosexuals: Results from the 2010 National HIV Behavioral Surveillance Study. http://www1.nyc.gov/assets/doh/downloads/pdf/dires/nhbshet2_may2011.pdf. Accessed: December 9 2016.

35. Diaz, T., et al., *Injection and syringe sharing among HIV-infected injection drug users: implications for prevention of HIV transmission. Supplement to HIV/AIDS Surveillance Group.* J Acquir Immune Defic Syndr Hum Retrovirol, 1998. **18 Suppl 1**: p. S76-81.

36. Schlumberger, M.G., et al., *Knowledge of HIV serostatus and preventive behaviour among European injecting drug users: second study. European Community Study Group on HIV in Injecting Drug Users.* Eur J Epidemiol, 1999. **15**(3): p. 207-15.

37. Rhodes, T.J., et al., *Continued risk behaviour among HIV positive drug injectors in London: implications for intervention.* Addiction, 1993. **88**(11): p. 1553-60.

38. Marks, G., et al., *Meta-analysis of high-risk sexual behavior in persons aware and unaware they are infected with HIV in the United States: implications for HIV prevention programs.* J Acquir Immune Defic Syndr, 2005. **39**(4): p. 446-53.

39. Leichliter, J.S., et al., *Temporal trends in sexual behavior among men who have sex with men in the United States, 2002 to 2006-2010.* J Acquir Immune Defic Syndr, 2013. **63**(2): p. 254-8.

40. Latkin, C.A., et al., *Norms, social networks, and HIV-related risk behaviors among urban disadvantaged drug users.* Soc Sci Med, 2003. **56**(3): p. 465-76.

41. Wejnert, C., et al., *HIV infection and awareness among men who have sex with men-20 cities, United States, 2008 and 2011.* PLoS One, 2013. **8**(10): p. e76878.

42. Galai, N., et al., *Longitudinal patterns of drug injection behavior in the ALIVE Study cohort,1988-2000: description and determinants.* Am J Epidemiol, 2003. **158**(7): p. 695-704.

43. Lieb, S., et al., *Statewide estimation of racial/ethnic populations of men who have sex with men in the U.S.* Public Health Rep, 2011. **126**(1): p. 60-72.

44. Hamilton, D.T., M.S. Handcock, and M. Morris, *Degree distributions in sexual networks: a framework for evaluating evidence.* Sex Transm Dis, 2008. **35**(1): p. 30-40.

45. Kaplan, E.H., *Modeling HIV infectivity: must sex acts be counted?* J Acquir Immune Defic Syndr, 1990. **3**(1): p. 55-61.

46. Fu, T.C., et al., *Changes in sexual and drug-related risk behavior following antiretroviral therapy initiation among HIV-infected injection drug users.* AIDS, 2012. **26**(18): p. 2383-91.

47. Marshall, B.D., et al., *No evidence of increased sexual risk behaviour after initiating antiretroviral therapy among people who inject drugs.* AIDS, 2010. **24**(14): p. 2271-8.

48. Kuyper, L., et al., *Does initiation of HIV antiretroviral therapy influence patterns of syringe lending among injection drug users?* Addict Behav, 2011. **36**(5): p. 560-3.

49. Singh, B.K., et al., *Sexual risk behavior among injection drug-using human immunodeficiency virus positive clients.* Int J Addict, 1993. **28**(8): p. 735-47.

50. Tun, W., et al., *Increase in sexual risk behavior associated with immunologic response to highly active antiretroviral therapy among HIV-infected injection drug users.* Clin Infect Dis, 2004. **38**(8): p. 1167-74.

51. Hollingsworth, T.D., R.M. Anderson, and C. Fraser, *HIV-1 transmission, by stage of infection.* J Infect Dis, 2008. **198**(5): p. 687-93.

52. Gomez, G. and S.W. Lagakos, *Estimation of the infection time and latency distribution of AIDS with doubly censored data.* Biometrics, 1994. **50**(1): p. 204-12.

53. Celentano, D.D., et al., *Time to initiating highly active antiretroviral therapy among HIV-infected injection drug users.* AIDS, 2001. **15**(13): p. 1707-15.

54. Uhlmann, S., et al., *Methadone maintenance therapy promotes initiation of antiretroviral therapy among injection drug users.* Addiction, 2010. **105**(5): p. 907-13.

55. Morris, J.D., et al., *Injection drug use and patterns of highly active antiretroviral therapy use: an analysis of ALIVE, WIHS, and MACS cohorts.* AIDS Res Ther, 2007. **4**: p. 12.

56. Zaccarelli, M., et al., *Factors related to virologic failure among HIV-positive injecting drug users treated with combination antiretroviral therapy including two nucleoside reverse transcriptase inhibitors and nevirapine.* AIDS Patient Care STDS, 2002. **16**(2): p. 67-73.

57. Kerr, T., et al., *Determinants of HAART discontinuation among injection drug users.* AIDS Care, 2005. **17**(5): p. 539-49.

58. Kaplan, E.H. and R. Heimer, *A model-based estimate of HIV infectivity via needle sharing.* J Acquir Immune Defic Syndr, 1992. **5**(11): p. 1116-8.

59. Baggaley, R.F., et al., *Risk of HIV-1 transmission for parenteral exposure and blood transfusion: a systematic review and meta-analysis.* AIDS, 2006. **20**(6): p. 805-12.

60. Bangsberg, D.R., et al., *Adherence to protease inhibitors, HIV-1 viral load, and development of drug resistance in an indigent population.* AIDS, 2000. **14**(4): p. 357-66.

61. Vittinghoff, E., et al., *Per-contact risk of human immunodeficiency virus transmission between male sexual partners.* Am J Epidemiol, 1999. **150**(3): p. 306-11.

62. Royce, R.A., et al., *Sexual transmission of HIV.* N Engl J Med, 1997. **336**(15): p. 1072-8.

63. Baggaley, R.F., R.G. White, and M.C. Boily, *HIV transmission risk through anal intercourse: systematic review, meta-analysis and implications for HIV prevention.* Int J Epidemiol, 2010. **39**(4): p. 1048-63.

64. Gray, R.H., et al., *Probability of HIV-1 transmission per coital act in monogamous, heterosexual, HIV-1-discordant couples in Rakai, Uganda.* Lancet, 2001. **357**(9263): p. 1149-53.

65. Quinn, T.C., et al., *Viral load and heterosexual transmission of human immunodeficiency virus type 1. Rakai Project Study Group.* N Engl J Med, 2000. **342**(13): p. 921-9.

66. Little, S.J., et al., *Viral dynamics of acute HIV-1 infection.* J Exp Med, 1999. **190**(6): p. 841-50.

67. Bangsberg, D.R., et al., *Non-adherence to highly active antiretroviral therapy predicts progression to AIDS.* AIDS, 2001. **15**(9): p. 1181-1183.

68. Egger, M., et al., *Prognosis of HIV-1-infected patients starting highly active antiretroviral therapy: a collaborative analysis of prospective studies.* Lancet, 2002. **360**(9327): p. 119-29.

69. May, M., et al., *Prognosis of HIV-1-infected patients up to 5 years after initiation of HAART: collaborative analysis of prospective studies.* AIDS, 2007. **21**(9): p. 1185-97.

70. Hudgens, M.G., et al., *Subtype-specific transmission probabilities for human immunodeficiency virus type 1 among injecting drug users in Bangkok, Thailand.* Am J Epidemiol, 2002. **155**(2): p. 159-68.

71. Williams, B.G., R. Granich, and C. Dye, *Role of acute infection in HIV transmission.* Lancet, 2011. **378**(9807): p. 1913; author reply 1914-5.

72. Bellan, S.E., et al., *Reassessment of HIV-1 acute phase infectivity: accounting for heterogeneity and study design with simulated cohorts.* PLoS Med, 2015. **12**(3): p. e1001801.

73. Eaton, J.W. and T.B. Hallett, *Why the proportion of transmission during early-stage HIV infection does not predict the long-term impact of treatment on HIV incidence.* Proc Natl Acad Sci U S A, 2014. **111**(45): p. 16202-7.

74. Windrum, P., G. Fagiolo, and A. Moneta, *Empirical validation of agent-based models: alternatives and prospects.* JASSS - J Artif Soc S, 2007. **10**(2): p. 8.

75. Xia, Q., et al., *Proportions of patients with HIV retained in care and virally suppressed in New York City and the United States: higher than we thought.* J Acquir Immune Defic Syndr, 2015. **68**(3): p. 351-8.

76. Marshall, B.D.L., et al., *Prevention and treatment produced large decreases in HIV incidence in a model of people who inject drugs.* Health Aff (Millwood), 2014. **33**(3): p. 401-9.

77. Marshall, B.D.L., et al., *A complex systems approach to evaluate HIV prevention in metropolitan areas: Preliminary implications for combination intervention strategies.* PLoS ONE, 2012. **7**(9): p. e44833.
